# DroughtCast: A Machine Learning Forecast of the United States Drought Monitor

**DOI:** 10.3389/fdata.2021.773478

**Published:** 2021-12-21

**Authors:** Colin Brust, John S. Kimball, Marco P. Maneta, Kelsey Jencso, Rolf H. Reichle

**Affiliations:** ^1^ Numerical Terradynamic Simulation Group, W.A. Franke College of Forestry and Conservation, University of Montana, Missoula, MT, United States; ^2^ Regional Hydrology Lab, Geosciences Department, University of Montana, Missoula, MT, United States; ^3^ Department of Ecosystem and Conservation Sciences, W.A. Franke College of Forestry and Conservation, University of Montana, Missoula, MT, United States; ^4^ Montana Climate Office, W.A. Franke College of Forestry and Conservation, University of Montana, Missoula, MT, United States; ^5^ Global Modeling and Assimilation Office, NASA Goddard Space Flight Center, Greenbelt, MD, United States

**Keywords:** machine learning, drought, recurrent neural network, U.S. drought monitor, forecasting, SMAP

## Abstract

Drought is one of the most ecologically and economically devastating natural phenomena affecting the United States, causing the U.S. economy billions of dollars in damage, and driving widespread degradation of ecosystem health. Many drought indices are implemented to monitor the current extent and status of drought so stakeholders such as farmers and local governments can appropriately respond. Methods to forecast drought conditions weeks to months in advance are less common but would provide a more effective early warning system to enhance drought response, mitigation, and adaptation planning. To resolve this issue, we introduce DroughtCast, a machine learning framework for forecasting the United States Drought Monitor (USDM). DroughtCast operates on the knowledge that recent anomalies in hydrology and meteorology drive future changes in drought conditions. We use simulated meteorology and satellite observed soil moisture as inputs into a recurrent neural network to accurately forecast the USDM between 1 and 12 weeks into the future. Our analysis shows that precipitation, soil moisture, and temperature are the most important input variables when forecasting future drought conditions. Additionally, a case study of the 2017 Northern Plains Flash Drought shows that DroughtCast was able to forecast a very extreme drought event up to 12 weeks before its onset. Given the favorable forecasting skill of the model, DroughtCast may provide a promising tool for land managers and local governments in preparing for and mitigating the effects of drought.

## Introduction

Drought is one of the most pervasive natural disasters affecting the United States. A single drought event can cause more than one billion dollars in damages and lead to the shift or degradation of entire ecological regimes (Crausbay et al., 2017; Smith 2020). Intensification of warm and dry meteorological anomalies across the country has strained crops and pastures (Boyer et al., 2013; Li et al., 2019), accelerated the spread of invasive pests and pathogens (Jactel et al., 2012), and driven extreme wildfire conditions, causing more frequent and severe wildfires than at any point in the last 2000 years (Holden et al., 2018; Higuera et al., 2021). Despite the broad socioeconomic and ecological impacts, the onset, extent, and duration of drought are difficult to define because different stakeholders have varying degrees of tolerance and resilience to these events (Slette et al., 2019). For example, meteorological, ecological, agricultural, hydrologic, and socio-economic droughts are all caused by a different combination of environmental and economic factors, making it difficult to create a single holistic definition of drought (Wilhite and Glantz 1985; IPCC, 2021). A further complication is the recent emergence of flash drought in the literature (e.g., Mo and Lettenmaier 2015; Otkin et al., 2018; Chen et al., 2019; Pendergrass et al., 2020). Flash droughts are characterized by their rapid onset, which tends to be driven by anomalously high temperatures, high evapotranspiration (ET), low precipitation, and low soil moisture (Otkin et al., 2018). Although less common than typical droughts, flash droughts can pose a significant risk, as they have driven widespread crop and livestock losses leading to notable economic and ecological damage (Otkin et al., 2018; He et al., 2019).

Several methods exist to monitor the status and progression of drought. For example, the Evaporative Demand Drought Index, Palmer Drought Severity Index, and Standardized Precipitation Evapotranspiration Index are common indices that use precipitation and ET data to estimate the intensity of hydrological and meteorological drought (Palmer 1965; Vicente-Serrano et al., 2010; Hobbins et al., 2016). In the U.S., one of the most popular means of monitoring drought is the United States Drought Monitor (USDM). The USDM uses a combination of meteorological data and expert opinion to produce weekly maps of categorical drought severity for the U.S., ranging from D0 (abnormally dry) to D4 (exceptional drought; Svoboda et al., 2002). While these indices are useful for monitoring the current status of drought, they do not provide information about future drought conditions. The ability to better forecast drought conditions weeks to months in advance would give stakeholders greater lead time in planning, preparing, and allocating critical resources for more effective drought mitigation. Further, the ability to forecast drought even a week in advance could significantly improve flash drought response, given their characteristic rapid onset (Otkin et al., 2018; Pendergrass et al., 2020). While there is no agreed upon definition of flash drought, it is becoming a common research topic and has many working definitions (Lisonbee et al., 2021). For example, studies have defined flash drought as rapid changes in the USDM that are sustained for four or more weeks (e.g., Chen et al., 2019; Pendergrass et al., 2020), while others define it as rapidly evolving anomalies in soil moisture (e.g., Liu et al., 2020; Sehgal et al., 2021) or evapotranspiration (Christian et al., 2019).

Methods for effective drought forecasting are possible because future drought status correlates with antecedent soil moisture, ET, and meteorological conditions. For example, anomalous decreases in soil moisture are generally reflected in degraded vegetation greenness and productivity weeks to months after soil drying occurs (Liu et al., 2011, 2016). Therefore, signs of drought may not become apparent until long after drought conditions begin. For example, Liu et al. (2011) found that when soil moisture reaches below normal conditions, it takes between 10 and 20 days for this decline to reflect in the plant production. Similarly, Liu et al. (2016) found that relationships between soil moisture and vegetation leaf area are significantly correlated up to 2 months after soil moisture anomalies begin. This lagged vegetation response to drought conditions is also outlined in Otkin et al. (2018), which shows that when paired with above average atmospheric vapor pressure deficit (VPD) and below average precipitation, the following three conditions can precede the onset of a drought: decreasing soil moisture content due to enhanced ET; decreasing ET due to low root zone soil moisture; deteriorating vegetation and ecological health.

Several existing methods exploit these leading indicators to forecast changes in drought conditions in the coming weeks and months. For example, NOAA’s National Center for Environmental Information (NCEI) and Climate Prediction Center (CPC) produce large scale maps of drought improvement or degradation for 1 month lead times, based upon “subjectively derived probabilities guided by short- and long-range statistical and dynamical forecasts.” However, these models do not forecast the potential for drought across the USDM severity levels (e.g., D0-D4). In addition, they are based upon meteorological forecasts and do not account for land surface interactions such as antecedent soil moisture conditions. Similarly, Otkin et al. (2014) developed a “Rapid Change Index” that uses anomalies in ET to detect regions of the contiguous United States (CONUS) where the USDM status will intensify. Finally, Lorenz et al. (2017a) used anomalies in precipitation, soil moisture, and ET to detect CONUS regions where the USDM is most likely to intensify. While these products are extremely useful in forecasting drought, they exhibit one or more of the following drawbacks:

1) They do not directly translate to a USDM category (i.e., they predict that drought will change but not *how much* drought will change). This particularly raises problems in forecasting rapid onset flash droughts, as a flash drought can be defined as a 2-category increase in the USDM that is sustained for 2 more weeks (Pendergrass et al., 2020);
2) They only provide forecasts at fixed intervals of 2, 4 and 8 weeks into the future, limiting the ability to track the possible progression of a drought at finer time scales;
3) They only use input variables that are traditionally assumed to correlate to future drought conditions such as precipitation, ET, and soil moisture. While these variables indeed correlate to future drought conditions, additional variables such as temperature, atmospheric humidity, and wind speed may contribute value-added information.

A promising approach to forecast USDM categories is through machine learning (ML). ML is becoming more common in earth system modeling and provides a unique means for making a prediction using complex, non-linear interactions of geospatial variables (Reichstein et al., 2019). Additional benefits of ML models include their ability to discover subtle or hidden patterns in complex geospatial data, and to map variables to an output without any a priori knowledge of how these variables interact (Reichstein et al., 2019). Specifically, recurrent neural networks (RNNs) and convolutional neural networks (CNNs) are becoming popular for extracting temporal and spatial relationships, or patterns, from observational input data and predicting a desired output. For example, Fang et al. (2017) used a variety of meteorological data in an RNN framework to accurately predict soil moisture observed from the NASA SMAP (Soil Moisture Active Passive) satellite over the CONUS domain. Additionally, Zhang et al. (2018) used a CNN to fill gaps in MODIS (Moderate Resolution Imaging Spectroradiometer) satellite imagery that were missing or degraded due to sensor errors or cloud cover. Recently, modeling frameworks combining these two approaches have been developed to account for both temporal and spatial context when making a prediction. A compelling example from Chao et al. (2018) combines an RNN and a CNN to accurately forecast precipitation at relatively fine temporal and spatial resolutions.

To address gaps in drought forecasting methods and exploit the power of ML models, this study introduces DroughtCast: an ML model framework trained to forecast future USDM categories up to 12 weeks into the future using satellite observed and modeled meteorological input features. The objectives of this study are to 1) assess the accuracy of the model framework and determine its ability to make accurate forecasts in years and spatial locations where it was not trained; 2) investigate the impact and importance of the remote sensing-based and modeled meteorological inputs on model forecasts of the USDM; and 3) examine model forecasts of the 2017 US Northern Plains flash drought as a regional case study to quantify how well the model performs in an extreme flash drought event. The following sections describe the study area and datasets used to train and run the model, as well as the model architecture and training and validation process (*Materials and Methods* Section); a summary of model performance and accuracy (*Results* Section); implications and significance of model results (*Discussion* Section); and the major conclusions of the study (*Conclusion* Section).

## Materials and Methods

### Study Area

The study area for this paper covers the entire CONUS from June 2003 to January 2020, which is the largest area and period covered simultaneously by all model inputs. The CONUS study domain is defined by the input feature record used for model training. The CONUS domain also spans a wide gradient in landcover, climate aridity, and terrain types, making it an ideal region for developing and testing robust drought forecast methods.

### Model Inputs

All features used to train and run our model forward are summarized in Table 1. Model training features are a combination of satellite observed and modeled meteorological variables that have been identified as key predictors of drought in previous studies (Otkin et al., 2018; Pendergrass et al., 2020). All model input features are clipped to the CONUS domain and projected to the SMAP 9-km cylindrical equal area (EASE-2) earth grid (Brodzik et al., 2014) using bilinear interpolation, as the SMAP data has the coarsest spatial resolution of the remote sensing-based model input features. USDM data are provided in a vector shapefile format, which are rasterized to match the data type of the other model inputs (Ross 2020). The rasterized USDM maps were then used as both a model input feature and training data in a recurrent, auto-regressive model. Because USDM data are available on a weekly basis, all daily or sub-daily datasets are converted to a weekly timestep by taking the mean of all data for a given dataset within a week; monthly datasets are linearly interpolated to a weekly timestep, and annual features are held constant throughout a forecast based on the calendar year that the forecast begins (i.e., if a forecast begins in December of 2017, annual inputs from 2017 are used for the entire forecast even though much of the forecast takes place in 2018). To balance the magnitude of model inputs and facilitate model training, all inputs are converted to normalized anomalies between −1 and 1, calculated as:

$$\begin{array}{c} \mathrm{normalized}=2*\frac{x-\min\left(x\right)}{\max\left(x\right)-\min\left(x\right)}-1 \end{array}$$ (1)

where *x* is the value of a given pixel, *min(x)* is the minimum value across the domain for the period of record, and *max(x)* is the maximum value across the domain for the study period.

**TABLE 1:** Model inputs used to train and run the DroughtCast model.

Product	Description	Spatial resolution	Temporal resolution	Features	References
U.S. Drought Monitor	Weekly drought status used as an input feature and model training data	∼10 km × ∼10 km	Weekly	- USDM	Svoboda et al. (2002)
SMAP Level-4 Soil Moisture	3-hourly surface (0–5 cm depth) and rootzone (0–1 m depth) estimates of soil moisture	9 km × 9 km	3-h	- Surface Soil Moisture	Reichle et al. (2019)
				- Rootzone Soil Moisture	
SMAP Level-4 Carbon	Daily estimates of GPP calculated using a SMAP water supply constraint	9 km × 9 km	Monthly*	- Gross Primary Production	Jones et al. (2017)
					Endsley et al. (2020)
MCD12Q1	Annual land cover	500 m	Annual	- Land cover	Friedl et al. (2002)
SMAP ET	Monthly estimates of ET calculated using a SMAP soil moisture constraint	9 km × 9 km	Monthly	- Evapotranspiration	Brust et al. (2021)
gridMET	Daily meteorology interpolated from local weather stations	4 km × 4 km	Daily	- Minimum temperature	Abatzoglou (2013)
				- Maximum temperature	
				- Minimum relative humidity	
				- Maximum relative humidity	
				- Vapor pressure deficit	
				- Precipitation	
				- Wind speed	
				- Solar radiation	
Multivariate ENSO Index	Index quantifying the strength of the El Niño Southern Oscillation	N/A	Monthly	- MEI	Wolter and Timlin (2011)

*Note: The SMAP L4_C is a daily product, but here we are using the monthly bias-corrected version.

A combination of 15 features derived from the remote sensing observations and modeled meteorology products listed in Table 1 were used as training features for DroughtCast. The USDM drought maps were used as both target classes and input features to provide the model with antecedent information on drought condition prior to producing a forecast (Svoboda et al., 2002). We use estimates of surface (0–5 cm) and rootzone (0–1 m) soil moisture from version 4 of the SMAP Level-4 soil moisture product (L4SM; Reichle et al., 2019). The L4SM system produces these soil moisture estimates by assimilating low frequency (L-band) microwave brightness temperature observations from the SMAP satellite into the GEOS-5 Catchment Land Surface Model (CLSM; Koster et al., 2000; Ducharne et al., 2000; Reichle et al., 2019), which is driven by surface meteorological data from the NASA Goddard Earth Observing System (GEOS) weather analysis (Lucchesi 2018). Model inputs of vegetation gross primary production (GPP) are obtained from the SMAP Level-4 Carbon product (L4C), which uses MODIS vegetation observations along with SMAP L4SM soil moisture and temperature data, and GEOS surface meteorology as inputs to a terrestrial carbon flux algorithm to provide daily estimates of GPP and net ecosystem CO_2_ exchange globally (Jones et al., 2017). We further use ET data derived from a modified MODIS MOD16 algorithm that incorporates SMAP L4SM soil moisture as a water supply constraint (Mu et al., 2011; Brust et al., 2021).

Because the SMAP satellite was launched in 2015, SMAP data are only available from March 31, 2015, to present. However, “NatureRun” (NR) versions of all SMAP products are available from the beginning of our study period through March 30, 2015. These NR products use the CLSM without the assimilation of SMAP observations to run the models forward. Although the NR accuracy is lower than the operational versions of these products, which benefit from incorporating SMAP observations, the NR error is still low over the CONUS domain, so they are used here when SMAP data are not available (Reichle et al., 2019; Endsley et al., 2020; Brust et al., 2021). There is a small bias between the NR and operational L4C product climatologies. To fix this issue, we use the bias correction method described in Wurster et al. (2021) and Endsley et al. (2020) to align the L4C operational and NR climatologies.

Estimates of daily maximum and minimum relative humidity, vapor pressure deficit, maximum and minimum air temperature, wind speed, precipitation, and solar radiation from the gridMet dataset (Abatzoglou 2013) are used as meteorological inputs for DroughtCast. Finally, we use a monthly Multivariate El-Niño Southern Oscillation Index (MEI) as a long-term climatic indicator (Wolter and Timlin 2011). In addition to these model inputs, several other ancillary variables that do not change over the course of a forecast are used as static inputs. These include the annual MODIS MCD12Q1 landcover product (Friedl et al., 2002), elevation provided from the gridMet dataset (Abatzoglou 2013), the day-of-year the forecast is made from, and pixel-wise averages across the study period of all previously mentioned model input features.

### Model Architecture

**FIGURE 1:**
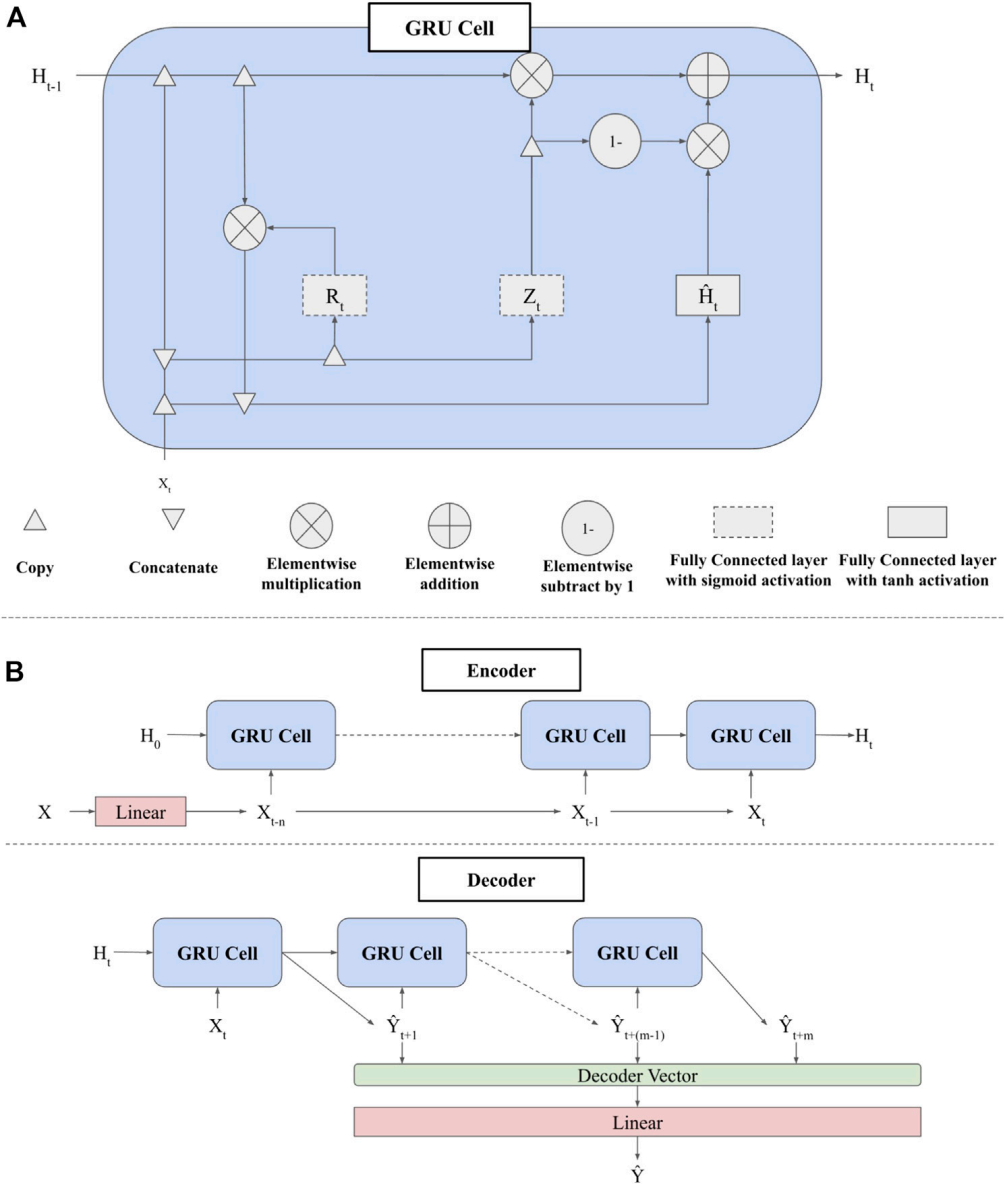
**(A)** Architecture of a single Gated Recurrent Unit (GRU) cell, recreated from Zhang et al. (2020). H_t_ is the hidden state vector for time step t; R is the reset gate, which computes values of H that should be forgotten; Z is the update gate, which computes values of H that should be remembered; and $\tilde{H}$ is the candidate hidden state, which has irrelevant values from H removed by the reset gate. **(B)** The full architecture of DroughtCast. In the encoder, the hidden state is sequentially updated with model inputs X. The final hidden state is then passed into the decoder, which produces a sequence of outputs. These outputs are then passed through a linear neural network to produce a timeseries of drought forecasts.

To forecast changes in the USDM, we use a Seq2Seq model architecture (Sutskever et al., 2014). The Seq2Seq architecture is designed to take sequential data as an input (e.g., a spatially distributed timeseries of meteorology and hydrology; *Model Training* Section) and produce a sequential output (e.g., a timeseries forecast of the USDM). The Seq2Seq architecture consists of an encoder and a decoder. The encoder processes a sequence of features and compresses it into a single context vector, which is then passed to the decoder. The decoder then uses the context vector to sequentially produce model outputs. In DroughtCast, the model encoder and the decoder are both gated recurrent units (GRUs; Figure 1A; Chung et al., 2014; Zhang et al., 2020). The GRU uses a timeseries of input features to sequentially update a hidden state vector, a compressed representation of all previously observed input features. There are three steps required to calculate the hidden state at time *t*. First, input features at time *t* and the hidden state from time *t-1* are used to derive the reset and update gates:

$$\begin{array}{c} R_{t}=\sigma \left(X_{t}W_{\mathrm{xr}}+H_{t-1}W_{\mathrm{hr}}+b_{r}\right) \end{array}$$ (2)

$$\begin{array}{c} Z_{t}=\sigma \left(X_{t}W_{\mathrm{xz}}+H_{t-1}W_{\mathrm{hz}}+b_{z}\right) \end{array}$$ (3)

where *R*_*t*_ is the reset gate, *Z*_*t*_ is the update gate, σ is a sigmoid function that transforms inputs between 0 and 1; *X*_*t*_ are input features at time *t*; H_t-1_ is the hidden state from the previous timestep; *W*_*xr,*_ *W*_*hr,*_ *W*_*xz,*_ *W*_*hz*_ are the input and hidden state weight matrices for the reset and update gates, respectively; and b_r_ and b_z_ are the bias vectors for the respective reset and update gates. The reset gate determines values in the hidden state that should be forgotten, while the update gate determines values in the hidden state that should be remembered. The reset gate is then used to calculate the candidate hidden state:

$$\begin{array}{c} {\tilde{H}}_{t}=\tanh\left(X_{t}W_{\mathrm{xh}}+\left(R_{t}\circ H_{t-1}\right)W_{\mathrm{hh}}+b_{h}\right) \end{array}$$ (4)

where $\tilde{H}$ is the candidate hidden state; *tanh* is the hyperbolic tangent function, which transforms inputs between −1 and 1; and ∘ denotes elementwise (Schur) multiplication. The candidate hidden state contains new hidden state values with irrelevant values removed from the reset gate. The candidate hidden state is finally combined with the results of the update gate to produce the hidden state at time t:

$$\begin{array}{c} H_{t}=Z_{t}\circ H_{t-1}+\left(1-Z_{t}\right)\circ {\tilde{H}}_{t} \end{array}$$ (5)

The DroughtCast architecture consists of the encoder and decoder GRUs and a simple neural network (NN) before the encoder and after the decoder (Figure 1B). Each of these NNs are comprised of a series of linear, dropout, batch normalization, and ReLU non-linearity layers (Srivastava et al., 2014; Ioffe and Szegedy 2015). These layers prevent overfitting and help the model generalize to data it has not seen before. The NN before the encoder is meant to build a more complex representation of input features, while the NN after the decoder takes the final hidden state and converts it into a single drought forecast value between 0 and 1. Forecasted values are then used with the corresponding USDM values to calculate error and update model parameters (*Model Training* Section). Rather than treating the USDM as an ordinal variable ranging from D0–D4, we treat it as an integer variable ranging from 0 (no drought) to 5 (D4—exceptional drought). These integer values are then divided by five (i.e., the total number of defined categories) to scale them to real numbers between 0 and 1, the same range as the forecasts produced by the DroughtCast model. While the USDM drought status is provided as an ordinal variable, we convert the USDM values to the continuous scale because we found that model performance is significantly improved when producing continuous rather than categorical forecasts (not shown). To convert model outputs to spatially continuous maps of the domain, model forecasts are produced on a pixel-by-pixel basis, starting at the top left corner of the domain, and moving to the bottom right corner. Once forecasts are produced for all pixels in the domain, the vector of forecasts are reshaped to match the dimensions of the domain.

Finally, we apply one final function to the modeled results from DroughtCast:

$$\begin{array}{c} f\left(x\right)=\{\begin{array}{c} \mathrm{round}\left(x*5\right),\;\;x\leq 0.6\\ \mathrm{ceil}\left(x*5\right),x>0.6 \end{array} \end{array}$$ (6)

where *f(x)* is the modeled forecast for a given pixel containing forecasts from 1–12 weeks into the future; *x* is the output produced by the neural network; *round* is a function rounding *x* to the nearest integer and *ceil* is a function returning the ceiling integer of *x* (e.g., if *x* equals 4.1, *ceil* returns 5). The above Equation 6 partially accounts for more severe (category D3 and D4) droughts being comparatively rare in the model training record, which can impart a forecast bias toward more frequent, but less severe drought categories. For example, the USDM states that for a given location and year, a D3 drought should occur between 3–5% of years, and a D4 drought should occur in less than 2% of years (Svoboda et al., 2002). Due to this scarcity, the model sees relatively few training samples of these drought classes which may degrade forecast performance for these more extreme events. The post-processing function simply rounds-up all model forecasts ranging between 3 and 5 to their nearest integer value. This processing step was found to significantly improve model performance in both training and test sets, while adding very little complexity or additional computational burden to the model architecture.

### Model Training

To train the model, we used a hidden state with 128 parameters, as a hyperparameter grid search found that these values produced optimal model forecasts (not shown). To update model parameters, a minibatch size of 128 pixels across all USDM images are randomly selected. For a given USDM image at date *d*, we then sampled the same set of pixels for all input features with dates ranging from *d-30 weeks* to *d-1 week*, as well as all USDM targets from dates *d* to *d+11 weeks*. Using a hyperparameter search, we found a 30-weeks history to provide favorable results while allowing the model to train in a reasonable amount of time. In each minibatch, DroughtCast produces 12 forecasts for each sampled pixel. These leading forecasts are then compared to the ground truth USDM images from 1–12 weeks into the future using the mean squared error (MSE) loss function. After the loss is computed, model parameters are updated *via* the backpropagation algorithm. This process is repeated until all possible pixel-image combinations are exhausted. This training loop is repeated 50 times or until the loss in the validation dataset stabilizes and stops decreasing. Data from selected years 2007, 2014, and 2017 were excluded from this process and used as holdout cross-validation datasets. We selected these holdout years to represent documented drought anomalies, including a large drought that occurred in the southeastern CONUS in 2007; the midpoint (2014) of a long-term drought extending across California, and the Northern Plains Flash Drought that occurred in 2017. This left 698 unique USDM images that were sampled and used for model training. The model training was performed on an NVIDIA Tesla P4 GPU and took approximately 2 weeks. After the model was trained, producing a forecast for the CONUS on a given day takes approximately 1 min on the same GPU.

We trained 10 of the models described above to produce an ensemble of model forecast estimates. Parameter updates, as well as the parameters that are turned off in the dropout layers, are not deterministic, meaning two models trained on the same data could produce slightly different results. Therefore, the model ensemble accounts for the stochastic nature of the machine learning model training and predictions. Pixel-wise median, maximum, minimum, and inter-quartile range (IQR) summary statistics were subsequently calculated from the model ensemble for each grid cell and time step. These summary statistics were then used to evaluate model performance and uncertainty in all analyses described below. All of the following error statistics and maps are derived using the ensemble median unless otherwise noted.

### Model Validation

#### Spatial and Temporal Generalization Tests

To ensure DroughtCast does not overfit to the training data, we performed two separate validation tests. The first test is a spatial generalization test; whereby, model training and test data are split into distinct CONUS sub-regions. Supplementary Figure S1 displays the CONUS sub-regions used for model training and testing. In both the training and test sets, the MSE and the coefficient of determination (R^2^) were evaluated. The ability of the model to generalize to spatial regions where it was not trained was assessed by comparing MSE and R^2^ values between the training data and the spatial holdouts. In the second test, the MSE and R^2^ metrics were calculated for all model estimates occurring within the 2007, 2014, and 2017 holdout years. The model was not trained from these annual records, so favorable performance during the holdout years indicates that the model is able to generalize forecasts to years it was not trained on. Additionally, to ensure model forecasts are consistent with USDM classes, we produced confusion matrices of categorical model performance for the training, spatial holdout, and temporal holdout sets for all lead times.

#### Relative Importance of Model Inputs

To determine the relative importance of model inputs, we ran each ensemble member forward for the entire study period while iteratively replacing each of the inputs with uniformly random values between −1 and 1; i.e., the range each input is normalized to. We then calculated how much the model error changed when a given input was replaced with a random value. Finally, the model inputs were ranked according to their relative importance in the model forecast. Due to the computational resources needed to rerun this test for each ensemble member and each model input across the entire domain, we only performed this test for the gridMet and satellite-observed features. Similar to Lorenz et al. (2017a), preliminary analysis found that the antecedent USDM information was by far the most important feature for producing a forecast. Therefore, the USDM data were excluded in all model holdout test runs to avoid skewing the results.

#### Regional Case Study Forecast of the Northern Plains Flash Drought

In the summer of 2017, the states of Montana, Wyoming, North Dakota, and South Dakota experienced a severe flash drought, characterized by anomalously low precipitation, high temperatures, and high vapor pressure deficits (Otkin et al., 2018; He et al., 2019; Jencso et al., 2019; Hoell et al., 2020). In fact, large portions of these states had never experienced a category D3 or D4 drought during the period of record used for this study (Supplementary Figure S2). Consequently, our model was not trained on a set of data containing rare high severity drought conditions across this region. To assess model performance for this drought, we compared the ensemble forecasts to USDM maps at two different time periods across the drought’s progression, one before the emergence of D3 drought, and one before the emergence of the more severe D4 drought. A favorable model drought forecast that successfully captures the spatial and temporal progression, and severity of this extreme event would indicate that the model can produce reliable flash drought forecasts.

## Results

### Spatial and Temporal Generalization

**FIGURE 2:**
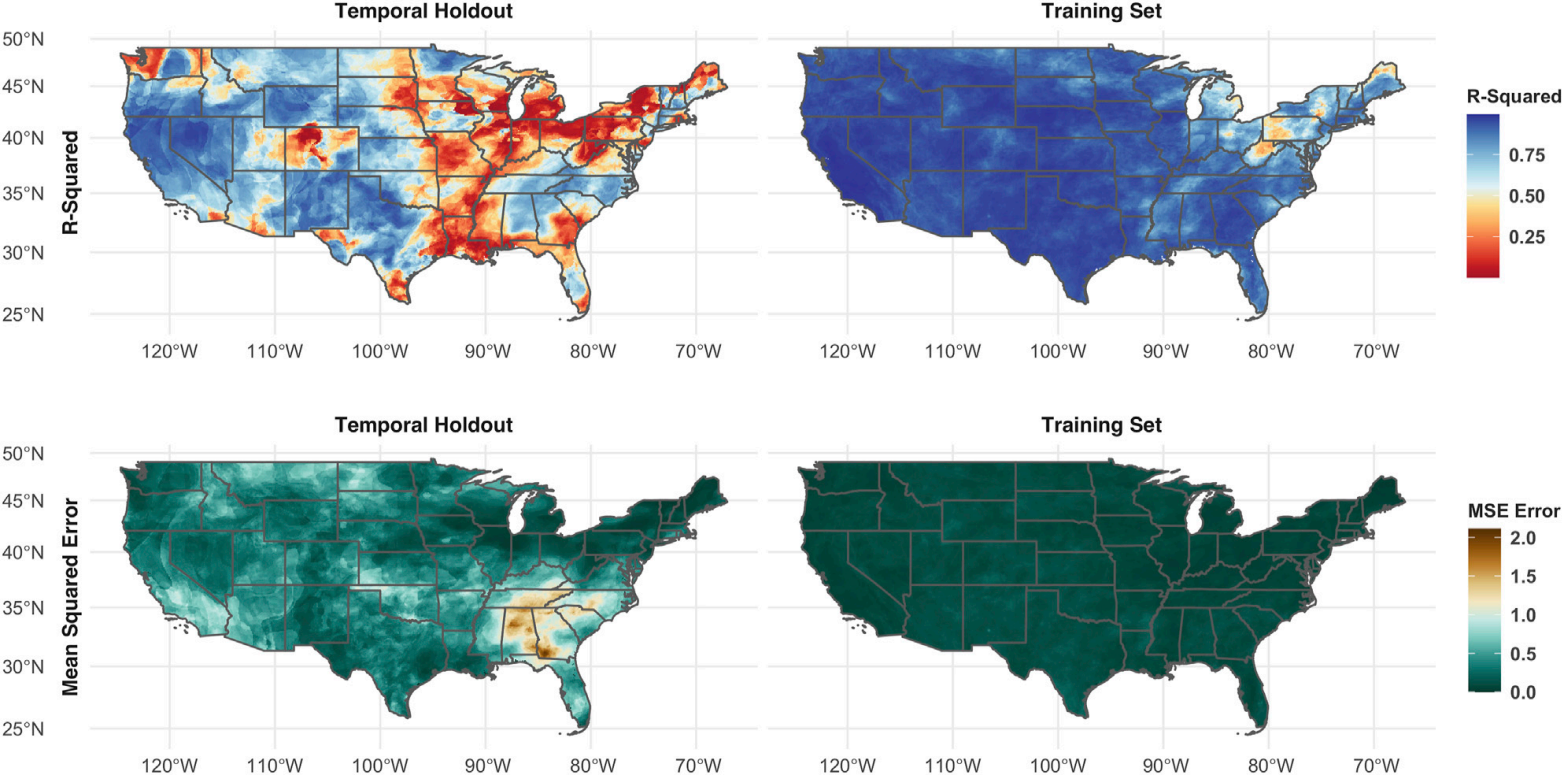
Spatial patterns of model MSE and correlation across the CONUS domain aggregated across all lead times. The top row shows the R^2^ correlation, while the bottom row shows the MSE error. The left column was calculated using data from 2007, 2014, and 2017, years that the model was not trained. The right column was calculated using data from years where the model was trained.

Across the entire domain, DroughtCast can accurately forecast USDM drought up to 12 weeks in advance (Table 2, Figure 2). Model forecast performance between the spatial holdout pixels is similar to the performance of the training pixels, while the performance in the temporal holdout data is slightly lower (Table 2). Spatially, this pattern is less consistent, as the temporal holdouts in the western CONUS have similar R^2^ and MSE performance relative to the training set, while the eastern CONUS has slightly degraded performance relative to the training set (Figure 2). Confusion matrices of categorical model performance show similar results (Supplementary Tables S1–S12). As in the MSE and R^2^ performance seen in Table 2 and Figure 2, the confusion matrices show that model performance is best at smaller lead times in the training set and spatial holdouts, and that misclassifications are more common in the temporal holdouts and as the lead time increases. In general, DroughtCast becomes less accurate for longer forecast lead times, particularly in the temporal holdout data. However, even in the model holdout years, the model forecast error at the maximum 12-weeks lead time is less than one USDM category and explains more than half (∼53%) of the variability in the USDM when aggregated across the CONUS domain (Table 2).

**TABLE 2:** Error and correlation for the DroughtCast ensemble at all lead times aggregated across the CONUS domain.

Lead time	MSE (USDM categories)			Correlation (R^2^)		
	Training data	Spatial holdout data	Temporal holdout data (2007, 2014, 2017)	Training data	Spatial holdout data	Temporal holdout data (2007, 2014, 2017)
1 Week	0.0534	0.0510	0.0567	0.9588	0.9575	0.9510
2 Week	0.0762	0.0753	0.1216	0.9411	0.9368	0.8955
3 Week	0.0870	0.0890	0.1823	0.9328	0.9253	0.8447
4 Week	0.0943	0.0992	0.2376	0.9270	0.9168	0.7994
5 Week	0.1003	0.1074	0.2862	0.9226	0.9099	0.7606
6 Week	0.1051	0.1139	0.3305	0.9185	0.9042	0.7259
7 Week	0.1093	0.1195	0.3717	0.9151	0.8993	0.6941
8 Week	0.1132	0.1247	0.4115	0.9118	0.8947	0.6642
9 Week	0.1171	0.1297	0.4503	0.9084	0.8902	0.6355
10 Week	0.1216	0.1353	0.4880	0.9048	0.8853	0.6081
11 Week	0.1267	0.1411	0.5232	0.9004	0.8798	0.5829
12 Week	0.1351	0.1493	0.5565	0.8934	0.8725	0.5597

Columns labelled “Train” are from all pixels used to train the model; columns labelled “Spatial” are pixels from the same years the model was trained, but from the spatial holdout set (Supplementary Figure S1); columns labelled “Temporal” are computed using all pixels from 2007, 2014, and 2017, years the model was not trained.

**FIGURE 3:**
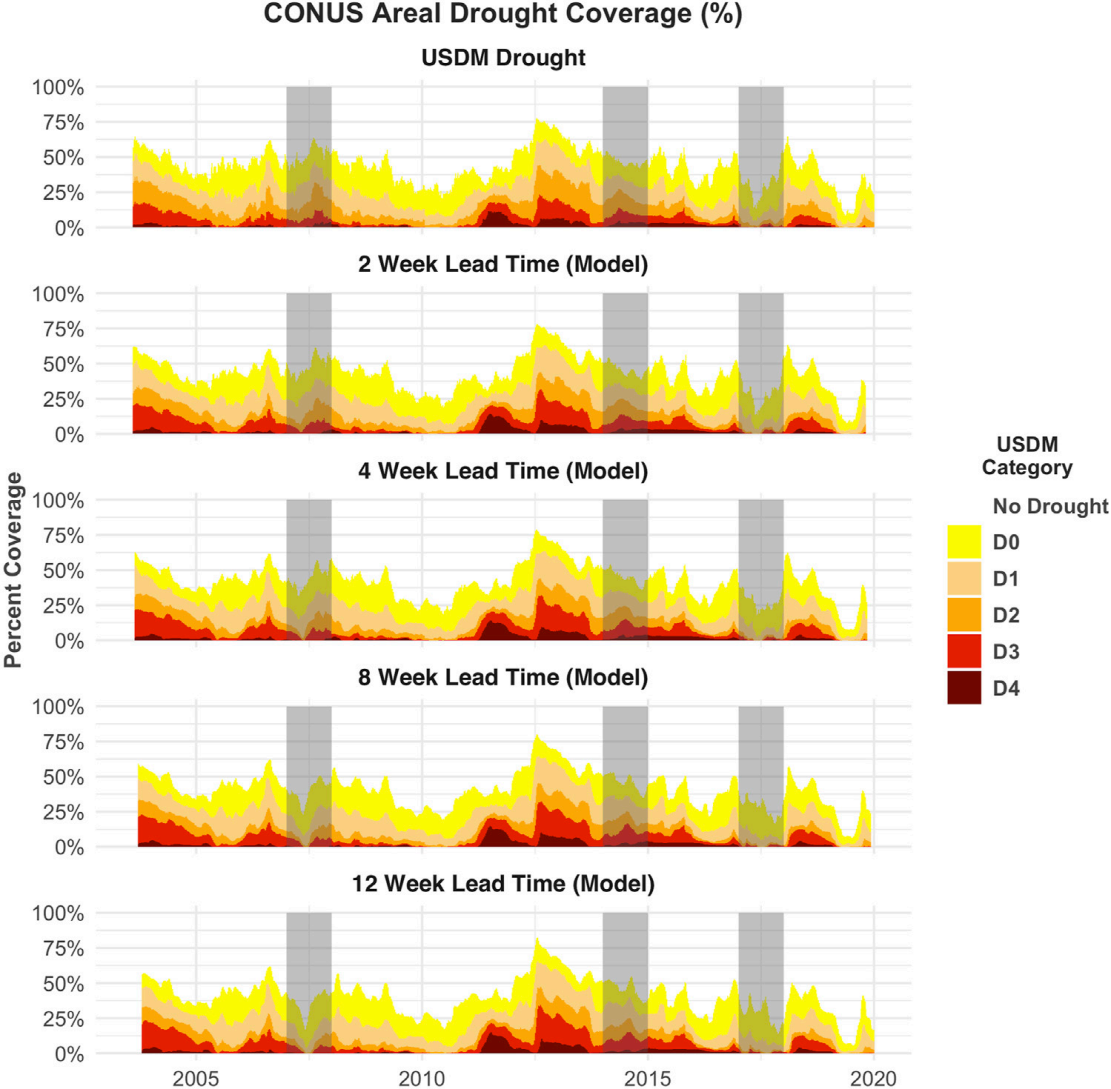
Areal coverage of drought categories across the CONUS for the study period. The top row shows the USDM drought, while the following rows show the forecasted distribution with 2-, 4-, 8-, and 12-weeks lead times, respectively.

**FIGURE 4:**
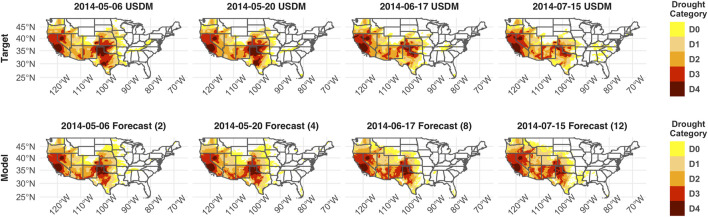
Example of model forecasting ability in a holdout year. This forecast was generated using the 30 weeks of data leading up to May 6th, 2014. Using this data, DroughtCast produces a forecast for each week, 1–12 weeks into the future. The top row shows the USDM drought, while the bottom row shows the model forecast for the same weeks, with lead times (2, 4, 8, 12 weeks) displayed in parentheses.

There are no clear regions of the CONUS where the model performs poorly in the training data. However, in the temporal holdout data, model MSE is notably degraded in California, Montana, and particularly in the Southeast (Figure 2). Despite the apparent degradation in these regions, the model R^2^ correlation remains high, suggesting that the model estimates may be consistently off by approximately one category in these regions (Figure 2). Despite these anomalies, the areal coverage of the estimated drought categories at all lead times closely matches that of the USDM areal coverage (Figure 3). In the holdout years, the model tends to underpredict high-intensity droughts (D3 and D4) and overpredict low-intensity (D0) droughts, particularly as the forecast lead time approaches 8–12 weeks. An example can be seen in Figure 4, where model forecasts at 8 and 12 weeks show expanded category D0 and D1 drought in much of Colorado, Wyoming, and Montana relative to the USDM. However, Figure 4 also illustrates the model’s ability to accurately track the intensification and reduction of drought. For example, the model captures the slight expansion of category D3 drought in California and Nevada, the persistence of D4 drought in California, and the shrinking of category D3 and D4 drought around Texas and Oklahoma.

The DroughtCast model ensemble successfully forecasts historical drought events such as the 2012 Central Plains drought, the dominant drought anomaly in the study record, across all lead times (Figure 3). Persistent, multi-year, severe drought over the southwestern CONUS throughout 2014 is also successfully forecasted at all lead times (Figures 3, 4). Additionally, the model forecasts are equally capable of detecting regions of the CONUS where drought does *not* occur (Figures 3, 4). Together, these patterns demonstrate the model’s capacity to capture different drought types occurring across diverse CONUS climate regimes.

### Relative Importance of Model Inputs

**FIGURE 5:**
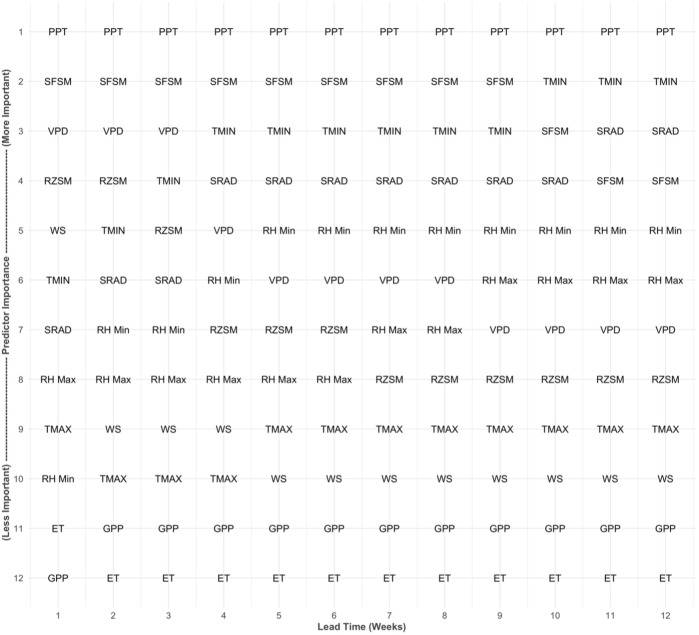
Relative importance of model input features by lead time. The y-axis represents the importance rank (1 = more important, 12 = less important), and the x-axis represents the forecast lead time (in weeks). The feature abbreviations are as follows: evapotranspiration (ET), gross primary production (GPP), precipitation (PPT), maximum relative humidity (RH Max), minimum relative humidity (RH Min), rootzone soil moisture (RZSM), surface soil moisture (SFSM), solar radiation (SRAD), maximum temperature (TMAX), minimum temperature (TMIN), vapor pressure deficit (VPD), wind speed (WS).

**FIGURE 6:**
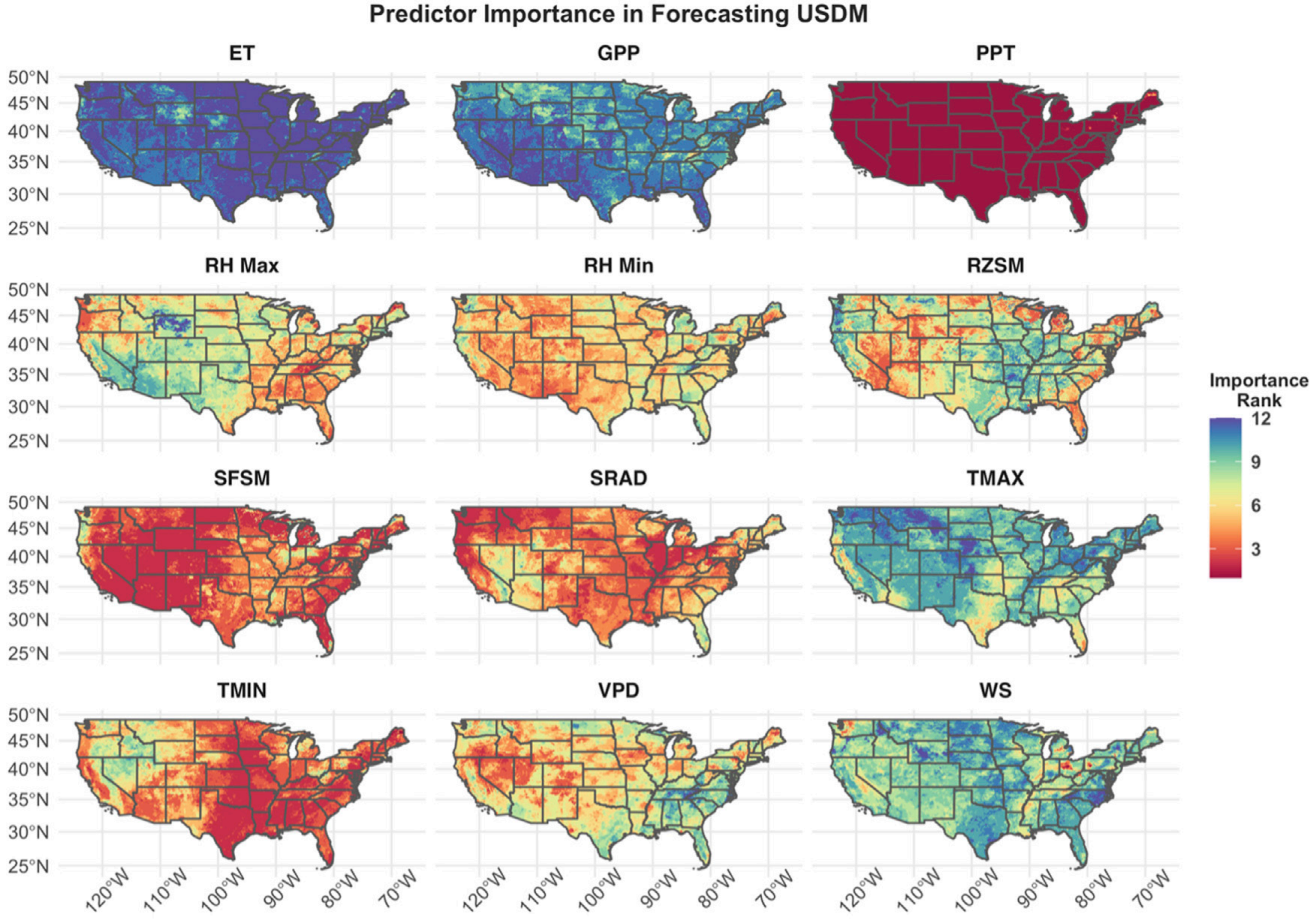
Relative importance of model input features by spatial location aggregated across all model lead times. Red colors represent higher input feature importance for a given area, while blue represents less important input features. The feature abbreviations are as follows: evapotranspiration (ET), gross primary production (GPP), precipitation (PPT), maximum relative humidity (RH Max), minimum relative humidity (RH Min), rootzone soil moisture (RZSM), surface soil moisture (SFSM), solar radiation (SRAD), maximum temperature (TMAX), minimum temperature (TMIN), vapor pressure deficit (VPD), wind speed (WS).

Across all lead times and the entire CONUS domain, precipitation is the most important input feature for the model USDM drought forecasts (Figures 5, 6). Of the satellite observed inputs, surface soil moisture and rootzone soil moisture are the most important predictors, respectively, while GPP and ET consistently rank lower (11th or 12th) in importance. However, the relative importance of the predictors shifts with longer lead times. For example, at lead times of 1, 2, and 3 weeks, VPD and rootzone soil moisture rank as the third and fourth most important input features, respectively (Figure 5). However, these features become less important at longer lead times. The opposite pattern is seen with minimum temperature and solar radiation. At 1-week lead time, minimum temperature and solar radiation are the sixth and seventh most important features, respectively, but they rise to become the second and third most important predictors by the 12-weeks lead time.

At all lead times, GPP, ET, maximum temperature, and wind speed rank among the least important features in forecasting future drought (Figure 5). Although these features rank poorly when aggregated across the entire domain, a map of feature importance across the CONUS shows that each of these features ranks higher in specific regions of the domain (Figure 6). For example, ET and GPP are approximately the seventh or eighth most important predictors in Montana and Wyoming, while maximum temperature and wind speed show similar ranking in California and the southeastern CONUS. Similar to the results displayed in Figure 5, precipitation is the most important feature for the entire CONUS domain, with minimum temperature, surface soil moisture, and solar radiation having similar, but secondary importance. While these features rank highly for the majority of the domain, their importance does vary between the eastern and western CONUS. For example, surface and rootzone soil moisture have greater ranking in the western CONUS and lower ranking in the eastern CONUS, while the opposite is true for solar radiation and minimum temperature.

### Northern Plains Flash Drought Case Study

**FIGURE 7:**
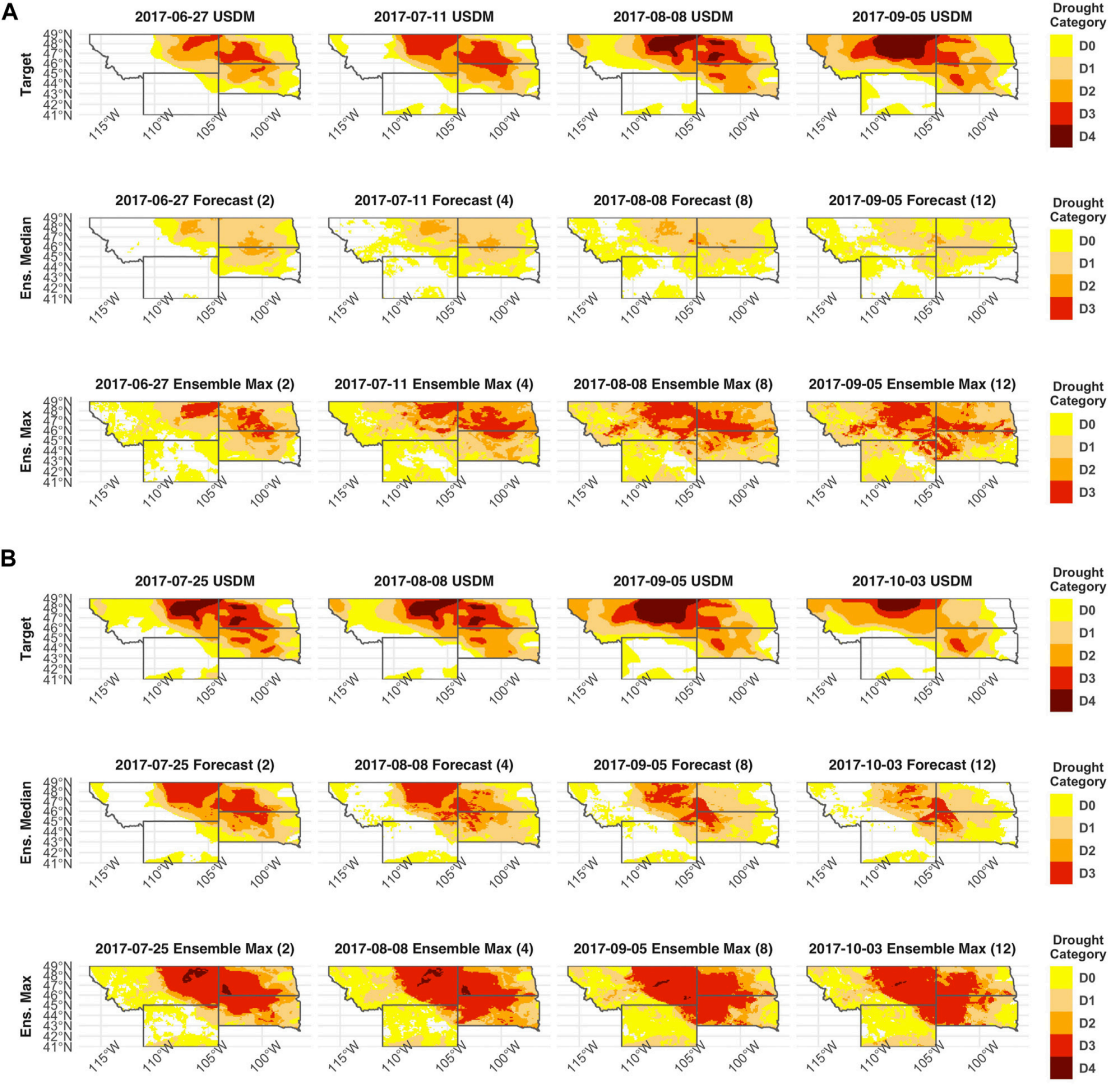
USDM and model forecasts of the 2017 Northern Plains Flash Drought. **(A)** Drought evolution early in the flash drought, with forecasts generated using data up to June 13th, 2017. **(B)** Drought evolution during the peak of the flash drought, with forecasts generated using data up to July 11th, 2017. In both figures, the first row shows the USDM on a given date, the second row shows the ensemble median, and the third row shows the ensemble maximum. The columns show the USDM and model forecasts at 2-, 4-, 8-, and 12-weeks lead times.

Anomalously dry and warm conditions in Montana, North Dakota, South Dakota, and Wyoming began to manifest in May and June of 2017 (He et al., 2019). Following these conditions, much of this region was under D0, D1 or D2 drought for much of May and June, and the first designation of category D3 drought in any of the states was on June 20th, 2017. Accordingly, our first forecast of the 2017 drought uses data up to June 13th to determine whether DroughtCast can properly capture the associated increase in drought intensity. Figure 7A shows the USDM status in a 12-weeks forecast, in addition to the DroughtCast ensemble median and maximum. The model median does not capture the emergence of category D3 drought except for a small portion of eastern Montana at the 8-weeks forecast mark. However, the median forecast does capture the expansion of drought conditions across the domain. While the ensemble median does not forecast the emergence of category D3 drought, the maximum of the ensemble does. At all forecast lead times, the ensemble maximum closely matches the USDM analysis of category D3 drought, but does not forecast the emergence of extreme D4 drought that emerges at the 8-weeks lead time mark (Figure 7A).

We produced another 12-weeks forecast after flash drought conditions began, but prior to the emergence of category D4 drought, which first appeared on July 18th, 2017. Accordingly, the resulting forecast uses input features leading up to July 11th. Here, the model median successfully captures the recession of category D3 drought across Montana and western North Dakota but fails to capture the emergence of category D4 drought (Figure 7B). The ensemble maximum, however, does capture the emergence and persistence of category D4 drought for the entire 12-weeks forecast, but does not forecast the D4 drought to be as spatially extensive as the USDM analysis. While neither the median nor maximums of the ensemble perfectly forecast the drought, a combination of the two provides a reliable estimate of where the flash drought both intensified and moderated over the course of the extreme event. For example, the ensemble median captured the maintenance of D1 and D2 drought in eastern Montana in the Dakotas throughout the forecast period, while the maximum captured the emergence of D4 drought prior to its designation by the USDM.

## Discussion

### Spatial and Temporal Generalization

The results from this study indicate that DroughtCast performs well from both the training data, as well as the spatial and temporal holdouts. However, the model results are better in the spatial holdouts relative to the temporal holdouts, which may be due to spatial autocorrelation in the input features and the relatively small sample size of the spatial holdouts relative to the training data. Because DroughtCast operates on a per pixel basis, it doesn’t account for interactions or similarity between adjacent pixels. Previous studies have used smoothed model inputs derived using a Gaussian kernel (Lorenz et al., 2017a) or used model architectures such as CNNs that account for spatial relationships between pixels (Chao et al., 2018). Despite this limitation, the spatial holdouts are still very accurate, including MSE performance within 1 USDM category and R^2^ correlation exceeding 50% for the longest (12-weeks) forecast lead time when aggregated across the CONUS domain.

Spatially, the model degradation in the temporal holdout tests was primarily located in the southeastern CONUS. This degradation coincides with an extreme drought event that occurred in the southeast in the spring and summer of 2007 (Lorenz et al., 2017a). Similar to the 2017 Northern Plains Flash Drought, this was a very extreme drought event with a magnitude that rarely occurred in the southeast for the remainder of the study period. For large portions of the southeastern states, the vast majority of category D3 and D4 drought to occur in the study period was in 2007 (Supplementary Figure S3), meaning the model saw relatively few severe category drought examples from this region in the training data. Additionally, the drought event was preceded by almost 2 years of less-than-normal precipitation, but the extensive D3 drought didn’t begin until almost a year after these anomalous conditions. Because these drought conditions did not begin until 2007, the model likely never effectively learned the underlying relationship between the preceding persistent low precipitation and subsequent high intensity drought in the CONUS southeast, resulting in a sub-optimal forecast of this extreme regional event. Despite the lower model performance in this region, the resulting MSE of the model forecast was still within two USDM categories in the southeast region (Figure 2).

### Relative Importance of Model Inputs

As expected, precipitation is the most important feature for all CONUS regions and across all lead times, followed by surface and root-zone soil moisture. Interestingly, rootzone soil moisture becomes a less important predictor at longer lead times, which contrasts with expectations of the slower evolving rootzone having longer soil moisture “memory” than the surface and being a better proxy for plant-available soil water (Reichle et al., 2019; Brust et al., 2021). As such, it is expected that rootzone soil moisture would be a more important feature in forecasting long term drought than surface soil moisture. However, Lorenz et al. (2017a) also found that surface soil moisture was slightly more important in forecasting future changes in the USDM than rootzone soil moisture. A possible explanation for this behavior in the current study is that the SMAP satellite only measures soil moisture in the top layer (∼0–5 cm depth) of the soil column, whereas the L4SM rootzone soil moisture is not directly observed by SMAP (Reichle et al., 2019), which may result in relatively less accurate L4SM rootzone estimates compared to the quality of the L4SM surface soil moisture. Moreover, there is also redundant information in the two features, where the model may recognize the redundancy and assign less weight to the rootzone soil moisture.

Redundant information in the model inputs may also account for the relatively low importance assigned to the ET and GPP inputs. Despite the high correlation between drought conditions and both GPP and ET (He et al., 2019), and the successful use of ET in other drought forecasting methods (e.g., Otkin et al., 2014), these features had relatively little influence within our modeling framework. Both ET and GPP are highly correlated with meteorological variables such as precipitation, solar radiation, soil moisture, and vapor pressure deficit (Peng et al., 2019; Brust et al., 2021). Therefore, the low importance of these parameters could reflect their high level of redundancy, with the model only using a small amount of the additional information that they carry. The lower GPP and ET importance may also reflect the coarse monthly aggregation of these parameters compared with the other input features delineated at weekly timescales. However, it is important to note that ET and GPP both contributed to the favorable model performance, particularly in the western CONUS (Figure 6).

### Forecast of Northern Plains Flash Drought

DroughtCast successfully captured the expansion and intensification of the 2017 flash drought, even though the 2017 holdout year was excluded from the model training. While the model ensemble median was less effective in forecasting the emergence of the D3 and D4 drought categories, the ensemble maximum forecast was more effective in predicting these regional drought extremes. The difficulty in forecasting these high category droughts is exacerbated by the paucity of D3 and D4 drought occurrence in these states during the study period. While portions of Montana, South Dakota, and Wyoming experienced severe D3 and D4 conditions during the historical 2012 Central Plains drought (Hoerling et al., 2014), these extremes were missing from other areas of Montana and North Dakota during the 2003–2020 study period (Supplementary Figure S2). Despite this limitation, the general pattern, magnitude, and progression of the 2017 flash drought were captured by the model ensemble maximum forecast, with generally better performance at shorter lead times and lower performance from the ensemble median forecast in representing the more extreme drought categories from this anomalous event. These results indicate that the model ensemble maximum may provide a more suitable USDM drought forecast given the projected intensification of climate extremes with global warming (IPCC, 2021), but it may also bias model forecasts toward greater drought extremes.

Despite never being trained on category D3 or D4 drought in northern Montana or North Dakota, the DroughtCast ensemble maximum forecasts effectively predicted these severe categories before their emergence. In addition to forecasting the emergence of D3 and D4 drought, the ensemble maximum model predictions forecasted a rapid 2-category intensification of the USDM within a 2-week period that persisted for another 2 weeks (Figure 7). The resulting pattern meets the flash drought criteria of USDM intensification proposed by Pendergrass et al. (2020) and Chen et al. (2019) and indicates that the DroughtCast ensemble maximum predictions successfully forecasted the 2017 Northern Plains Flash Drought.

In addition to the model’s ability to forecast this drought event, it should also be noted that the model forecasts accurately depict the intensification, mediation and decline of the USDM across the summer of 2017, consistent with the onset and amelioration of hydrologic and meteorological drought conditions. In the months leading up to June of 2020, the Northern Plains states experienced abnormally low precipitation and high temperatures, which drove rapid declines in soil moisture (Jencso et al., 2019). This led to the designation of D3 drought conditions in Montana and the Dakotas, which was effectively forecasted by DroughtCast (Figure 7). These conditions persisted through August, leading to the emergence of D4 drought, which was also captured by our ensemble maximum forecast. In August of 2017, the Dakotas experienced considerable precipitation, recharging the soil water supply, leading to the eventual decline in USDM drought severity (Jencso et al., 2019). A final forecast produced on August 29th, 2017, successfully forecasts the decline in USDM magnitude over the Dakotas, but the persistence of D3 and D4 drought over Montana, which did not experience any considerable precipitation through the month of August (Supplementary Figure S4).

### Model Uncertainties and Future Work

Despite favorable forecasting ability for much of the CONUS domain, the forecasts produced by DroughtCast have inherent uncertainty. Reasons for this uncertainty include the human component of the USDM, which involves a combination of meteorological and hydrologic data, and expert opinion (Svoboda et al., 2002). DroughtCast directly incorporates meteorological and hydrologic information, while the human element is only indirectly represented from the USDM data used for model training. The model framework may account for some of the uncertainty introduced by subjective expert opinion, but not all of it. However, as the USDM evolves to use more drought indicators and more robust drought monitoring methods are developed, our model can glean this information and continue to improve in its forecasting ability. Further, no set of meteorological or hydrological indicators will ever be able to fully capture the complex relationships between the biophysical changes that occur during a drought and the resulting effects on ecosystems and economies. This makes the subjectivity of the USDM essential, as it captures the regionalized impacts of drought that vary across the CONUS due to differences in economies, agriculture, and drought tolerance.

Variability in the resulting ensemble of forecasts (e.g., Figure 7) reflects the stochastic nature of the model training process. When training the model, we only updated model weights based on one minibatch representing 128 pixels of data at a time, and the dropout in the linear model layers is applied at random. As a result, the final model weights can vary based on the ordering of the data it is trained on and the random dropout. While this process can introduce uncertainty into a single model, the associated impact may be reduced by the ensemble of model projections, resulting in more robust model forecasts with up to 12-weeks effective lead times.

Another source of uncertainty is that the distribution of USDM categories is non-linear (i.e., the distance between D0 and D1 is not the same as the distance between D3 and D4; Lorenz et al., 2017b). We attempt to address this issue by implementing the rounding function outlined in Eq. 6. However, another option could be to calculate the cutoff threshold between USDM categories within our model (e.g., Beguería and Maneta, 2020). By training the model to calculate the points on the continuous scale where drought categories shift, we could forego the extra calculation outlined in Eq. 6 and potentially improve the model results. A final change to our model framework that could improve results is to better account for features with a strong seasonal cycle. Features such as temperature and solar radiation tend to have strong seasonality (i.e., low in the winter, high in the summer). As such, normalizing these features as we do in Eq. 1 does not provide context as to how the feature compares to previous years on a given date. Adding a seasonal average feature, rather than an annual average, as a model input may provide a more temporally explicit context for each of these features (Lorenz et al., 2017a).

The results presented here are meant to serve as a framework for future model refinements. A number of methods have recently been developed that could be used to improve on the results presented here. For example, the temporal fusion transformer (TFT) was recently developed as a means of forecasting timeseries data and implicitly accounting for model uncertainty (Lim et al., 2021). Additionally, a model framework that accounts for pattern recognition in both space and time could further improve model results. The framework implemented by Chao et al. (2018) would be a good option, as it uses an RNN architecture similar to that used in DroughtCast, but also implements spatial convolutions that account for spatial relationships between adjacent pixels. Additionally, improvements in the spatial resolution of the model inputs could lead to finer-scale drought forecasts. Here, we are limited to the relatively coarse resolution of the SMAP and USDM products. However, the potential exists for finer scale estimates of drought commensurate with finer scale model inputs (e.g., Chaney et al., 2016), enabling localized drought forecasts at the individual county or farm level to better inform risk management and mitigation efforts. Model forecasts could also be improved by using a wider variety and greater number of input features. For example, lower-order satellite observations such as Landsat reflectances (e.g., Ketchum et al., 2020; Moreno-Martinez et al., 2020) or SMAP brightness temperatures (e.g., Piepmeier et al., 2017; Tong et al., 2020) could be used as input features rather than modelled ET, GPP, and soil moisture. Recent studies have also found terrestrial water storage as measured by the GRACE satellites to be a useful drought metric (Zhao et al., 2017). Future studies could potentially use this data to improve modeled drought forecasts. Finally, dynamical subseasonal-to-seasonal (S2S) forecasts of precipitation and temperature (e.g., Hao et al., 2017) could be used as additional input features to provide the model with information about possible future meteorological conditions across the domain. Future model frameworks could be tested by forecasting more recent drought events in the CONUS such as the 2019 flash drought in the Southeast or the persistent drought over the West in 2020 and 2021.

## Conclusion

This paper introduced DroughtCast, an ML model that forecasts maps of USDM drought categories up to 12 weeks in advance. The model ensemble makes skillful predictions for years and regions where it was not trained, with an average error of less than one USDM drought category for a 12-weeks forecast in a holdout test dataset. Although our study is constrained to the CONUS domain, the model framework is flexible. Given sufficient data, the model framework could be deployed anywhere in the world that has a data repository of historic drought condition, high-resolution meteorology, and soil moisture. Our results show that of all model training features, precipitation, soil moisture, and temperature are key for forecasting drought. However, all features add value to the model forecasting ability and their relative importance varies across the CONUS. In a case study of the 2017 Northern Plains Flash Drought, DroughtCast successfully forecasted the emergence of category D3 and D4 drought, and the forecasts successfully met the definition of a flash drought.

Future studies can advance this framework by exploring finer spatial resolution training data, alternative ML, or deep learning model architectures, or expanded model input features such as S2S forecasts. Despite some shortcomings, DroughtCast has the potential to make operational drought forecasts that can be used by land managers, farmers, and government agencies to make informed and timely decisions about drought risk.

## Data Availability

The raw data supporting the conclusions of this article will be made available by the authors, without undue reservation.
