# Supplementary material for: DroughtCast: A Machine Learning Forecast of the United States Drought Monitor
Source: Front Big Data. 2021 Dec 21;4:773478. doi: 10.3389/fdata.2021.773478 (PMC8725730; doi:10.3389/fdata.2021.773478)
Supplement: Supplementary file 1 [file DataSheet1.pdf]

Supplementary Material

1 Model Spatial Training/Test Locations

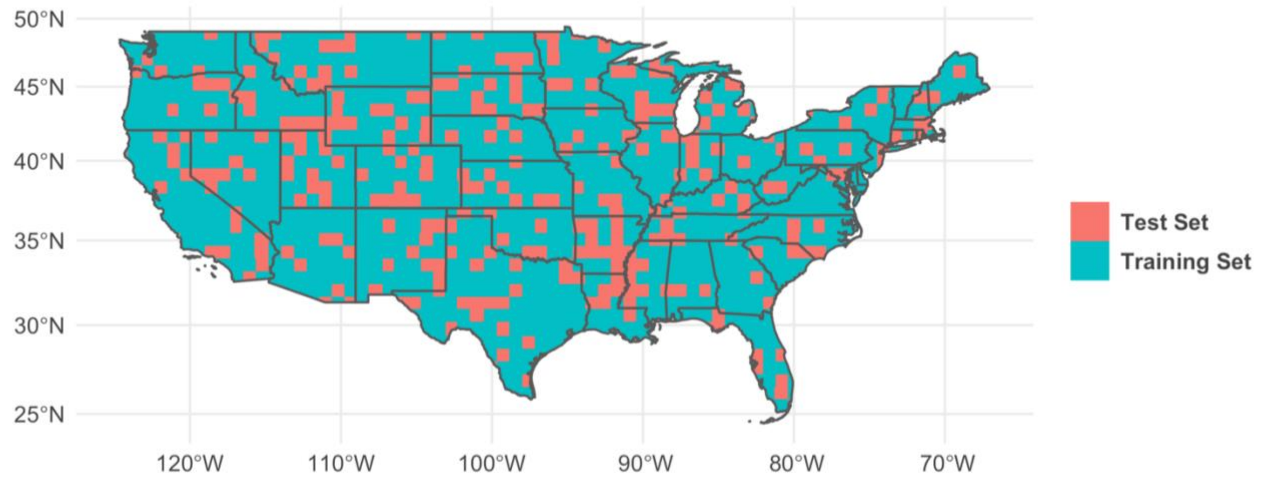

Supplementary Figure 1. Regions of the CONUS domain used to train (blue) and test (red) DroughtCast.

2 Frequency of High Intensity Drought Events Occurring in Model Holdout Years

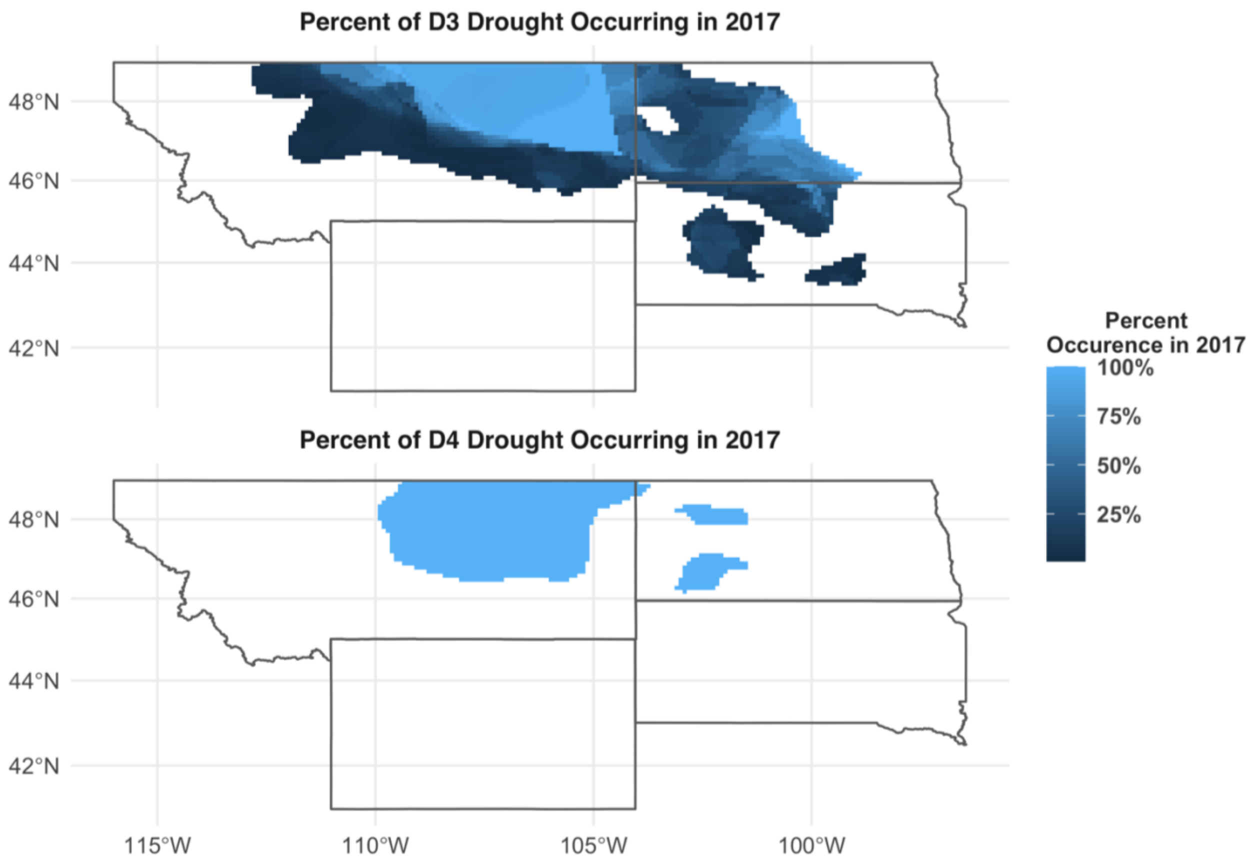

Supplementary Figure 2. The percentage of U.S. Drought Monitor category D3 and D4 drought that occurred in the 2017 holdout year relative to the 2003 – 2020 study record across the Northern Plains Flash Drought states.

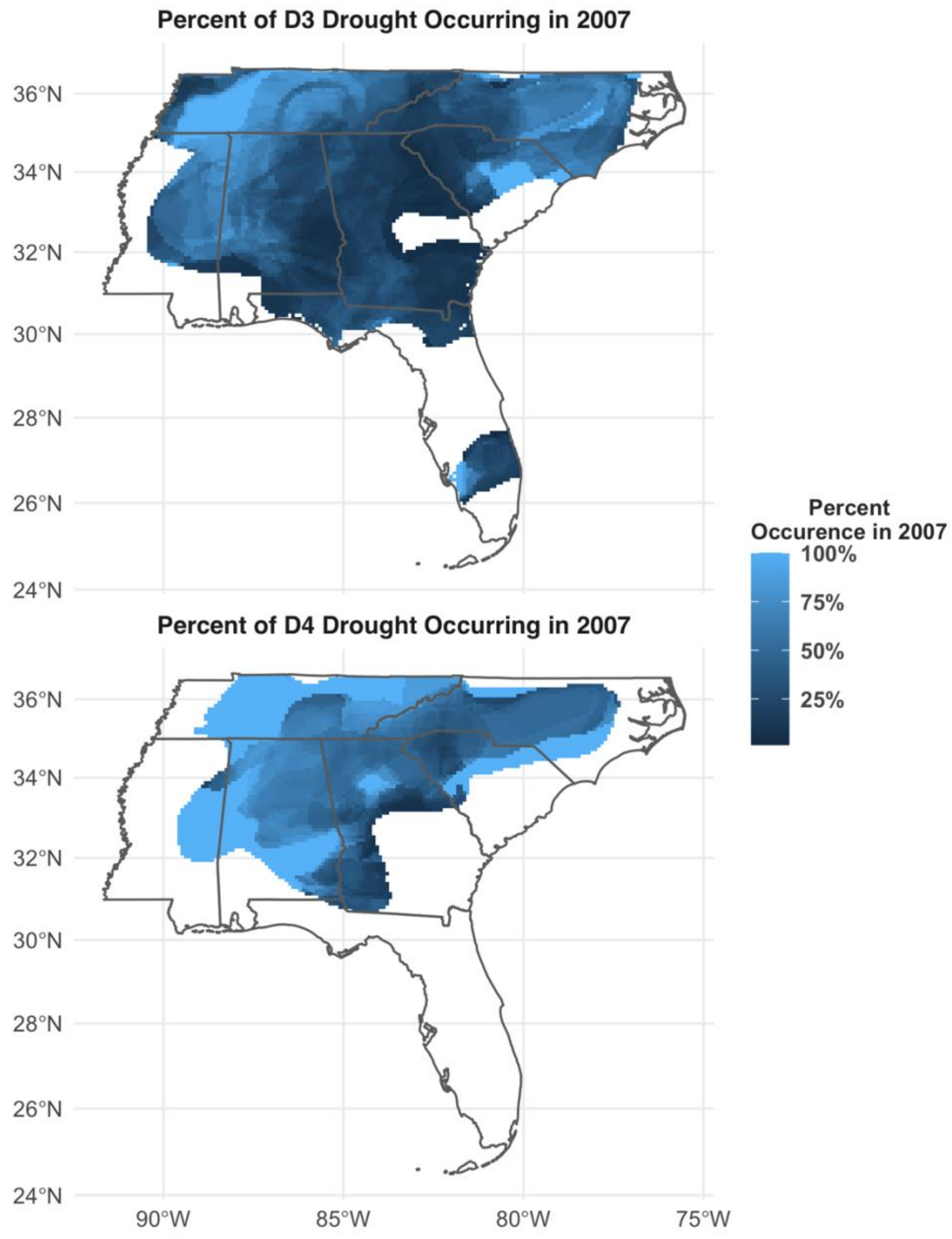

Supplementary Figure 3. The percentage of U.S. Drought Monitor category D3 and D4 drought that occurred in the 2007 holdout year relative to the 2003 – 2020 study record across the southeastern CONUS.

3 Model Forecast at the End of the 2017 Northern Plains Flash Drought

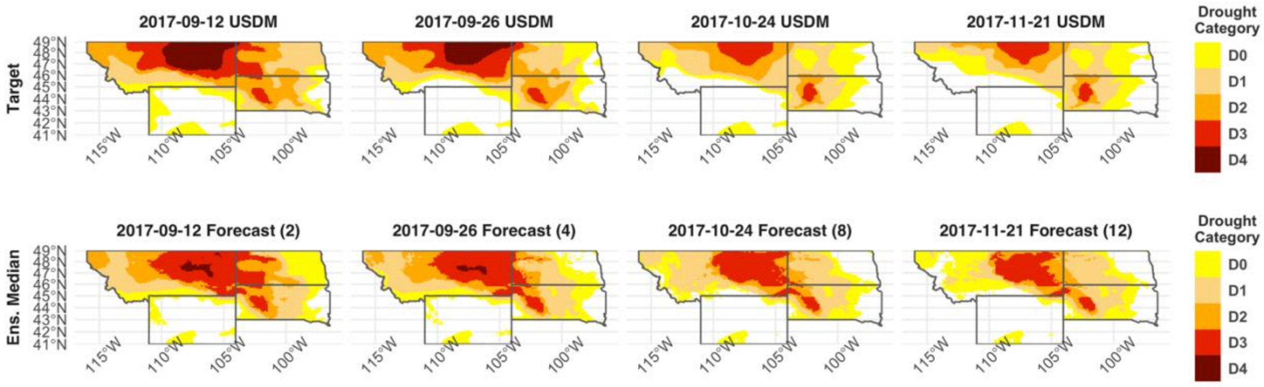

Supplementary Figure 4: DroughtCast forecast at the end of the 2017 Northern Plains Flash Drought using data leading up to August 29th, 2017, coinciding with the onset of precipitation in the Dakotas.

4 Confusion Matrices of Model Forecasts at Various Lead Times

Supplementary Table 1: Confusion matrix of pixel-wise categorical model performance for a 1-week lead time across the entire domain and study record.

1 Week Lead Time — Spatial Holdouts (rows: Predicted USDM Class; columns: True USDM Class)

Predicted \ True	No Drought	D0	D1	D2	D3	D4
No Drought	22,750,869	488,074	34,215	1,880	640	6
D0	521,431	1,976,769	236,243	15,528	2,102	24
D1	26,026	252,815	1,535,712	144,021	16,414	215
D2	179	9,698	178,308	956,283	267,407	4,636
D3	0	45	6,036	29,341	643,466	74,300
D4	0	0	20	21	16,014	247,837

1 Week Lead Time — Temporal Holdouts

Predicted \ True	No Drought	D0	D1	D2	D3	D4
No Drought	20,301,729	493,360	42,832	3,378	1,467	0
D0	550,849	1,820,558	194,599	15,439	3,451	108
D1	22,873	325,639	1,356,053	104,360	16,078	579
D2	190	21,864	189,784	1,059,680	115,752	4,073
D3	0	725	13,213	81,307	562,409	25,543
D4	0	3	226	1,637	53,222	174,108

1 Week Lead Time — Training Data

Predicted \ True	No Drought	D0	D1	D2	D3	D4
No Drought	66,364,656	1,555,162	103,925	5,418	1,113	6
D0	1,663,524	6,398,960	748,340	45,283	4,662	43
D1	82,736	801,575	4,921,303	457,618	46,496	592
D2	581	27,230	560,598	3,136,046	862,970	14,740
D3	7	370	17,753	89,364	2,191,834	235,278
D4	0	0	44	208	55,381	880,081

Supplementary Table 2: As in Table S1 but for a 2-week lead time.

2 Week Lead Time — Spatial Holdouts

Predicted USDM Class \ True USDM Class	No Drought	D0	D1	D2	D3	D4
No Drought	22,767,349	482,368	32,547	1,717	577	0
D0	647,105	1,821,017	272,758	11,312	1,794	0
D1	27,851	300,511	1,468,149	154,801	19,984	176
D2	406	10,551	234,537	856,970	305,315	5,079
D3	0	40	7,074	35,887	625,159	82,918
D4	0	0	0	95	20,034	242,494

2 Week Lead Time — Temporal Holdouts

Predicted USDM Class \ True USDM Class	No Drought	D0	D1	D2	D3	D4
No Drought	20,162,496	596,599	62,456	3,975	1,646	0
D0	864,342	1,482,189	219,085	16,438	4,712	22
D1	92,500	454,158	1,159,369	107,714	21,707	873
D2	5,754	63,520	293,579	878,969	148,596	5,209
D3	61	6,275	54,393	97,339	495,070	24,427
D4	0	188	1,526	7,579	71,343	152,979

2 Week Lead Time — Training Data

Predicted USDM Class \ True USDM Class	No Drought	D0	D1	D2	D3	D4
No Drought	66,473,803	1,494,279	85,336	3,475	863	0
D0	2,018,659	5,968,062	846,184	26,615	3,640	7
D1	80,401	933,272	4,755,525	474,599	55,358	420
D2	951	28,559	716,256	2,842,246	990,062	14,273
D3	10	123	20,858	103,242	2,141,526	264,603
D4	0	0	38	462	65,374	864,816

Supplementary Table 3: As in Table S1 but for a 3-week lead time.

3 Week Lead Time — Spatial Holdouts

Predicted USDM Class \ True USDM Class	No Drought	D0	D1	D2	D3	D4
No Drought	22,782,800	479,210	35,135	1,203	331	0
D0	714,273	1,729,117	296,378	10,990	1,766	0
D1	31,784	321,025	1,432,648	159,274	22,694	197
D2	636	12,533	263,510	797,764	328,037	5,283
D3	0	130	8,427	38,960	612,973	88,135
D4	0	0	1	122	23,663	237,576

3 Week Lead Time — Temporal Holdouts

Predicted USDM Class \ True USDM Class	No Drought	D0	D1	D2	D3	D4
No Drought	19,999,521	709,497	85,028	6,165	1,605	4
D0	1,079,605	1,245,308	244,275	20,255	4,991	197
D1	179,011	506,634	1,016,903	116,252	26,841	1,117
D2	21,568	102,453	336,769	776,895	159,835	5,969
D3	2,413	15,257	83,602	97,516	449,916	23,927
D4	0	497	5,215	13,413	78,140	140,494

3 Week Lead Time — Training Data

Predicted USDM Class \ True USDM Class	No Drought	D0	D1	D2	D3	D4
No Drought	66,588,653	1,426,966	79,823	2,623	815	0
D0	2,163,961	5,768,306	900,188	21,668	3,222	22
D1	81,963	976,076	4,699,777	472,327	57,660	413
D2	1,160	30,037	774,292	2,689,413	1,071,513	13,693
D3	20	168	21,037	113,807	2,110,052	278,459
D4	0	1	61	575	73,870	851,276

Supplementary Table 4: As in Table S1 but for a 4-week lead time.

4 Week Lead Time — Spatial Holdouts

Predicted USDM Class \ True USDM Class	No Drought	D0	D1	D2	D3	D4
No Drought	22,782,940	494,792	38,451	1,055	459	0
D0	759,594	1,658,378	317,301	10,615	2,285	5
D1	37,025	339,138	1,396,252	164,033	25,916	252
D2	881	14,639	280,542	750,836	349,569	5,725
D3	4	122	9,795	42,072	603,005	91,058
D4	0	0	12	301	26,380	233,143

4 Week Lead Time — Temporal Holdouts

Predicted USDM Class \ True USDM Class	No Drought	D0	D1	D2	D3	D4
No Drought	19,848,793	798,974	107,245	6,936	2,445	113
D0	1,214,481	1,113,173	255,193	22,890	5,806	337
D1	259,808	538,497	903,858	123,853	30,460	912
D2	42,828	132,479	358,400	695,839	177,452	4,956
D3	6,976	30,199	95,648	92,979	420,234	23,427
D4	13	914	11,244	16,683	80,157	132,886

4 Week Lead Time — Training Data

Predicted USDM Class \ True USDM Class	No Drought	D0	D1	D2	D3	D4
No Drought	66,634,068	1,435,821	81,532	2,485	879	1
D0	2,260,341	5,617,316	943,551	19,777	3,520	29
D1	90,191	1,028,618	4,617,786	475,915	61,705	418
D2	1,356	33,021	809,487	2,565,563	1,141,795	13,913
D3	35	290	22,642	118,733	2,088,675	283,384
D4	0	11	87	1,128	80,939	838,885

Supplementary Table 5: As in Table S1 but for a 5-week lead time.

5 Week Lead Time — Spatial Holdouts

Predicted USDM Class \ True USDM Class	No Drought	D0	D1	D2	D3	D4
No Drought	22,782,853	511,147	41,219	1,195	569	0
D0	795,633	1,605,395	328,038	11,614	2,668	8
D1	42,000	357,192	1,356,243	171,909	29,655	289
D2	1,108	16,945	292,406	713,873	366,737	6,159
D3	7	215	11,183	45,571	591,763	94,535
D4	0	1	47	450	28,898	229,050

5 Week Lead Time — Temporal Holdouts

Predicted USDM Class \ True USDM Class	No Drought	D0	D1	D2	D3	D4
No Drought	19,713,715	873,288	125,395	9,061	3,459	247
D0	1,306,917	1,031,743	261,186	24,041	6,930	314
D1	327,391	557,256	816,889	130,430	34,054	781
D2	65,511	159,436	366,576	633,091	191,081	5,125
D3	14,098	45,418	98,193	87,645	398,833	22,912
D4	59	1,565	17,251	18,737	81,961	126,499

5 Week Lead Time — Training Data

Predicted USDM Class \ True USDM Class	No Drought	D0	D1	D2	D3	D4
No Drought	66,659,933	1,459,523	84,635	2,625	1,185	1
D0	2,348,114	5,494,354	966,015	20,791	4,098	13
D1	96,688	1,081,812	4,521,225	493,501	68,028	362
D2	1,648	37,691	831,545	2,461,914	1,202,671	14,764
D3	30	465	26,142	121,297	2,063,939	292,469
D4	1	15	157	1,704	85,117	829,425

Supplementary Table 6: As in Table S1 but for a 6-week lead time.

6 Week Lead Time — Spatial Holdouts

Predicted USDM Class \ True USDM Class	No Drought	D0	D1	D2	D3	D4
No Drought	22,781,521	529,624	44,389	1,349	700	0
D0	819,155	1,564,625	338,755	12,557	3,120	8
D1	45,996	370,439	1,323,165	179,497	32,489	291
D2	1,267	19,188	302,903	689,547	372,220	6,692
D3	0	310	12,379	49,528	582,463	95,363
D4	0	3	130	497	30,945	225,460

6 Week Lead Time — Temporal Holdouts

Predicted USDM Class \ True USDM Class	No Drought	D0	D1	D2	D3	D4
No Drought	19,582,353	940,519	145,163	11,513	4,553	258
D0	1,371,734	976,414	268,061	25,643	7,870	295
D1	386,848	559,656	756,342	138,176	36,186	809
D2	86,805	188,803	366,141	585,970	196,007	5,262
D3	23,389	56,914	97,280	84,185	382,048	21,645
D4	209	3,360	22,888	20,907	81,292	121,590

6 Week Lead Time — Training Data

Predicted USDM Class \ True USDM Class	No Drought	D0	D1	D2	D3	D4
No Drought	66,667,733	1,498,382	91,171	3,077	1,397	1
D0	2,408,112	5,401,254	988,032	22,201	4,750	9
D1	102,467	1,116,136	4,445,240	511,685	72,224	382
D2	2,036	41,478	854,425	2,394,234	1,226,782	15,042
D3	31	810	29,147	126,886	2,043,772	293,482
D4	0	19	366	2,138	91,309	817,687

Supplementary Table 7: As in Table S1 but for a 7-week lead time.

7 Week Lead Time — Spatial Holdouts

Predicted USDM Class \ True USDM Class	No Drought	D0	D1	D2	D3	D4
No Drought	22,781,776	545,727	47,287	1,467	805	0
D0	837,919	1,530,316	348,296	13,326	3,401	10
D1	49,827	383,139	1,292,725	184,703	35,591	329
D2	1,504	21,576	314,544	665,066	377,282	7,360
D3	4	438	13,525	52,900	573,198	96,987
D4	0	4	156	528	32,420	222,439

7 Week Lead Time — Temporal Holdouts

Predicted USDM Class \ True USDM Class	No Drought	D0	D1	D2	D3	D4
No Drought	19,463,161	998,094	165,187	13,722	5,407	242
D0	1,419,373	935,063	274,974	27,783	8,971	230
D1	444,753	549,383	712,865	142,662	39,472	932
D2	108,530	215,290	361,677	547,661	198,042	5,576
D3	33,390	65,056	94,312	83,491	366,132	21,401
D4	736	6,736	26,459	23,450	79,308	117,567

7 Week Lead Time — Training Data

Predicted USDM Class \ True USDM Class	No Drought	D0	D1	D2	D3	D4
No Drought	66,667,365	1,540,296	96,322	3,650	1,540	0
D0	2,467,507	5,313,722	1,008,878	24,175	5,118	4
D1	108,727	1,154,793	4,368,719	524,348	77,434	449
D2	2,359	45,361	884,221	2,329,257	1,242,571	15,641
D3	30	1,325	32,705	132,368	2,019,413	298,704
D4	0	20	443	2,473	95,041	808,918

Supplementary Table 8: As in Table S1 but for an 8-week lead time.

8 Week Lead Time — Spatial Holdouts

Predicted USDM Class \ True USDM Class	No Drought	D0	D1	D2	D3	D4
No Drought	22,778,260	563,810	50,157	1,747	788	0
D0	850,187	1,504,291	357,144	14,609	3,643	14
D1	53,703	395,129	1,264,171	189,153	38,620	325
D2	1,787	24,090	324,613	645,524	379,011	7,444
D3	8	555	15,044	56,131	565,348	97,204
D4	0	8	188	615	34,478	218,776

8 Week Lead Time — Temporal Holdouts

Predicted USDM Class \ True USDM Class	No Drought	D0	D1	D2	D3	D4
No Drought	19,359,950	1,046,107	185,516	16,108	6,496	195
D0	1,458,772	892,533	283,922	31,525	9,599	210
D1	496,421	532,834	681,932	146,239	43,073	1,073
D2	131,913	235,393	356,342	517,788	196,801	5,734
D3	43,153	72,991	89,978	84,856	351,404	20,685
D4	2,638	9,475	29,524	25,370	77,692	112,846

8 Week Lead Time — Training Data

Predicted USDM Class \ True USDM Class	No Drought	D0	D1	D2	D3	D4
No Drought	66,657,833	1,587,022	102,247	4,090	1,601	4
D0	2,510,386	5,248,734	1,029,199	26,140	5,459	2
D1	115,764	1,187,735	4,297,212	536,591	81,365	425
D2	2,698	50,224	908,887	2,291,382	1,235,563	16,363
D3	54	1,742	36,286	141,049	1,997,882	296,857
D4	0	22	555	2,846	101,490	798,188

Supplementary Table 9: As in Table S1 but for a 9-week lead time.

9 Week Lead Time — Spatial Holdouts (rows: Predicted USDM Class; columns: True USDM Class)

Predicted USDM Class	No Drought	D0	D1	D2	D3	D4
No Drought	22,773,790	580,588	52,497	2,048	837	0
D0	866,900	1,478,049	362,781	16,111	3,911	20
D1	57,860	408,001	1,233,534	194,506	42,086	375
D2	2,016	27,561	331,697	626,429	382,915	8,015
D3	17	785	17,174	58,741	556,526	98,137
D4	0	27	192	670	36,348	215,431

9 Week Lead Time — Temporal Holdouts

Predicted USDM Class	No Drought	D0	D1	D2	D3	D4
No Drought	19,277,463	1,082,598	204,247	18,494	7,754	163
D0	1,494,538	848,395	294,059	35,811	10,656	194
D1	544,221	514,164	653,484	151,588	47,230	1,217
D2	157,726	251,760	345,601	488,644	198,319	5,761
D3	53,579	79,725	84,176	86,328	338,906	20,300
D4	4,661	12,437	33,232	24,601	76,525	108,531

9 Week Lead Time — Training Data

Predicted USDM Class	No Drought	D0	D1	D2	D3	D4
No Drought	66,647,892	1,631,378	108,196	4,416	1,790	0
D0	2,564,272	5,174,766	1,045,010	28,592	5,944	5
D1	123,713	1,227,607	4,217,167	549,245	87,063	414
D2	2,997	55,896	936,006	2,235,215	1,246,393	17,165
D3	71	2,301	40,267	149,470	1,972,196	298,412
D4	0	18	652	3,130	106,590	789,648

True USDM Class

Supplementary Table 10: As in Table S1 but for a 10-week lead time.

10 Week Lead Time — Spatial Holdouts

Predicted USDM Class \ True USDM Class	No Drought	D0	D1	D2	D3	D4
No Drought	22,764,816	598,869	55,811	2,560	938	0
D0	890,870	1,445,505	367,349	18,189	4,451	23
D1	63,515	424,692	1,198,961	199,235	46,565	441
D2	2,485	31,477	337,986	604,484	389,245	8,759
D3	28	1,093	18,994	61,762	546,428	99,803
D4	0	45	221	717	38,043	212,215

10 Week Lead Time — Temporal Holdouts

Predicted USDM Class \ True USDM Class	No Drought	D0	D1	D2	D3	D4
No Drought	19,209,979	1,104,628	222,209	20,819	9,509	159
D0	1,528,140	809,561	301,277	39,987	12,133	183
D1	587,679	496,972	626,322	156,041	51,061	1,232
D2	184,955	263,621	337,142	458,768	201,727	5,655
D3	65,252	84,296	78,428	86,219	330,736	20,164
D4	6,935	15,863	36,590	22,637	75,524	104,685

10 Week Lead Time — Training Data

Predicted USDM Class \ True USDM Class	No Drought	D0	D1	D2	D3	D4
No Drought	66,625,436	1,684,869	116,979	5,274	2,028	0
D0	2,639,787	5,078,161	1,058,866	32,453	6,723	1
D1	137,106	1,281,412	4,114,705	563,835	97,196	530
D2	3,599	63,367	963,181	2,164,063	1,268,219	19,008
D3	116	3,109	44,360	158,476	1,941,470	302,353
D4	0	26	805	3,380	112,456	780,548

Supplementary Table 11: As in Table S1 but for a 11-week lead time.

11 Week Lead Time — Spatial Holdouts

Predicted USDM Class \ True USDM Class	No Drought	D0	D1	D2	D3	D4
No Drought	22,745,878	623,078	62,986	3,295	802	1
D0	922,775	1,400,492	375,653	20,782	4,971	18
D1	72,128	439,167	1,164,057	204,532	50,434	522
D2	3,139	35,417	346,884	590,296	385,260	9,312
D3	40	1,507	22,033	66,899	534,759	99,449
D4	2	56	281	848	41,131	207,691

11 Week Lead Time — Temporal Holdouts

Predicted USDM Class \ True USDM Class	No Drought	D0	D1	D2	D3	D4
No Drought	19,152,680	1,118,301	242,934	23,167	11,250	125
D0	1,556,079	773,144	309,670	43,947	13,762	169
D1	623,252	484,340	602,229	161,819	52,383	1,318
D2	212,710	269,467	334,527	438,210	196,491	5,333
D3	78,186	84,325	75,473	88,169	319,789	19,759
D4	9,176	20,463	37,634	20,092	76,427	100,288

11 Week Lead Time — Training Data

Predicted USDM Class \ True USDM Class	No Drought	D0	D1	D2	D3	D4
No Drought	66,554,940	1,773,639	135,855	6,736	2,236	1
D0	2,755,488	4,919,438	1,091,404	37,736	8,037	13
D1	159,083	1,335,989	4,002,459	581,573	107,410	670
D2	4,459	74,066	1,009,496	2,107,645	1,251,927	21,052
D3	149	3,703	52,069	176,270	1,907,412	298,302
D4	1	38	1,014	3,795	123,653	766,139

Supplementary Table 12: As in Table S1 but for a 12-week lead time.

12 Week Lead Time — Spatial Holdouts

Predicted USDM Class \ True USDM Class	No Drought	D0	D1	D2	D3	D4
No Drought	22,721,309	649,930	71,697	3,912	1,114	2
D0	971,242	1,344,802	378,363	24,158	5,917	26
D1	86,603	460,154	1,113,755	210,452	56,226	667
D2	4,280	42,011	355,899	567,633	385,932	10,401
D3	127	2,087	25,611	72,474	522,048	98,856
D4	5	71	363	1,022	44,503	202,923

12 Week Lead Time — Temporal Holdouts

Predicted USDM Class \ True USDM Class	No Drought	D0	D1	D2	D3	D4
No Drought	19,113,821	1,125,158	258,655	25,305	13,023	115
D0	1,583,653	733,918	314,421	47,994	15,458	152
D1	654,568	476,345	576,767	168,529	54,449	1,361
D2	242,133	273,611	329,357	415,436	195,660	5,115
D3	91,492	82,343	74,754	88,856	310,372	18,859
D4	11,691	26,009	35,818	17,793	79,356	94,741

12 Week Lead Time — Training Data

Predicted USDM Class \ True USDM Class	No Drought	D0	D1	D2	D3	D4
No Drought	66,455,917	1,878,918	163,113	8,452	2,991	0
D0	2,933,715	4,706,088	1,114,106	47,979	10,125	40
D1	202,677	1,416,125	3,830,295	604,540	124,460	864
D2	6,246	93,959	1,055,806	2,020,794	1,253,750	24,648
D3	197	5,024	64,083	195,445	1,865,103	295,966
D4	22	66	1,298	4,888	136,886	749,311
